# Factors Associated with COVID-19 Testing, Vaccination, and Use of Digital Contact Tracing Apps among Black and Latinx MSM (BLMSM) in Los Angeles

**DOI:** 10.1007/s40615-023-01750-y

**Published:** 2023-08-11

**Authors:** Yan Wang, Raiza M. Beltran, William G. Cumberland, Sean D. Young

**Affiliations:** 1 Section of Public and Population Health, Division of Oral and Systemic Health Sciences, School of Dentistry, University of California, Los Angeles (UCLA), 10833 La Conte, Los Angeles, CA 90095 USA; 2 Luskin School of Public Affairs, UCLA, 10833 La Conte, Los Angeles, CA 90095 USA; 3 Department of Biostatistics, Fielding School of Public Health, UCLA, 10833 La Conte, Los Angeles, CA 90095 USA; 4 Department of Emergency Medicine, School of Medicine and Informatics, Information and Computer Sciences, University of California, Irvine, City Tower, Ste 640, Rt 128-01, Irvine, CA 92697 USA

**Keywords:** COVID-19, Minority, MSM, Test, Vaccine, Tracing app

## Abstract

This study examines the factors associated with COVID-19 testing, vaccination intent (both individually and jointly), and willingness to use contact tracing digital apps among a cohort of Black and Latinx men who have sex with men (BLMSM) living in Los Angeles during the initial peak (July 2020) of the COVID-19 pandemic. A questionnaire detailing participants COVID-19 experiences was sent to 300 primarily BLMSM after the first state-wide COVID-19 lockdown. Logistic regression models with random cluster effects were used for analyses. Forty-two percent (42%) tested for COVID-19, 27% were willing to get vaccinated, and about 45% reported willingness to use contact tracing digital apps. Controlling for intervention participation, age, education, marital status, employment, health, tobacco, binge drinking, and self-reported anxiety, those who were depressed had 33% (95% CI: 0.13 to 0.82) odds of using a prevention strategy (either test for COVID-19 or vaccination intent) as the group who were not depressed. Those who had high school diploma or less had 23% (95% CI: 0.11 to 0.48) odds to use digital contact tracing apps as the group with education level of at least Associate’s or Bachelor’s degree. Without considering the format of the test kits, vaccine side effects, and ease of use for digital contact tracing apps, participants appeared to still be hesitant in using COVID-19 prevention strategies at the initial height of the pandemic. Our findings suggest the need for further investigation into this hesitancy to better inform and prepare for future epidemics.

## Introduction

The first US COVID-19 case, caused by the severe acute respiratory syndrome coronavirus-2 (SARS-CoV-2), was detected in late January 2020 in the state of Washington, and the first reported COVID-19 community transmission occurred in California a week later, on January 26, 2020 [1]. California implemented a state-wide lockdown on March 20, 2020 [2]. Several virus transmission prevention strategies were implemented at the onset of the outbreak including mandatory mask-wearing in public, regular hand sanitizing, social distancing, and remote work. Once available, public health authorities also recommended regularly taking COVID-19 tests, getting two doses of the COVID-19 vaccines with boosters, and using a contact tracing app [3]. While these restrictions and regulations have been relaxed due to the effectiveness of the COVID-19 vaccine and booster doses, researchers are debating if this return to normalcy may be premature [4–6]. New variants of SARS-CoV-2 continue to be a threat while other infectious diseases that have long been endemic to certain areas have recently emerged as potential global pandemics—such as mpox that primarily affected men who have sex with men (MSM) in May 2022 [7]. Examining prevention strategies at the beginning of the COVID-19 pandemic, particularly among vulnerable populations such Black and Latinx men who have sex with men (BLMSM), is essential to maintain the health and wellbeing of this community [8].

### COVID-19 Health Disparities and Prevention Strategies among BLMSM

COVID-19–related health disparities persist in California where Latinx represent 54% of total COVID-19 cases but make up only 19% of the population [4]. Meanwhile, Black Californians have died of COVID-19 at a higher rate compared to their white peers [9–12]. Additionally, compared to their straight, cisgender, counterparts, sexual and gender minority (SGM) individuals of color were also found to be more likely to test positive for COVID-19 and were twice as likely to test positive than white-identified SGM [13]. Comorbid health conditions, such as cancer, heart disease, obesity, smoking, asthma, and diabetes, were also more prevalent and higher in severity among SGM individuals of color compared to their racially diverse non-SGM identified peers [14]. As of April 2022, nearly 71.7% of Californians were fully vaccinated, having received both shots of a two-dose regimen or a single dose. Still, Black and Latinx residents continue to have lower vaccination rates than other racial and ethnic groups, at 66% and 65% respectively [15].

Previous studies show that MSM report varying use of strategies for preventing infectious disease spread, despite their increased risk for certain vaccine preventable diseases such as human papillomavirus and invasive meningococcal disease [16, 17]. Reduced access to resources such as having suboptimal health insurance or lack of transportation to a nearby clinic [18], as well as experienced social stigma related to one’s sexual minority identity [19, 20] may have prevented gay and bisexual MSM from seeking preventive care. BLMSM face additional intersecting barriers related to their race and ethnicity. Structural and sociocultural barriers including historical mistrust of the health care system, immigration status, English language proficiency, health insurance availability, proximity to a health clinic, gender norms, and experienced homophobia within one’s culture were also associated with lower vaccination rates for BLMSM [21–23].

Since the COVID-19 pandemic, more focus has been placed on understanding vaccine hesitancy, particularly among marginalized populations [24, 25]. The “5C model” of vaccine hesitancy listed five person-level determinants: confidence, complacency, convenience, risk calculation, and collective responsibility [26]. Previous studies found that the vaccine acceptance rate was lower in the USA than in low- and middle-income countries [27, 28]. The factors affecting the hesitancy of receiving the COVID-19 vaccine were more complicated, however, such as low income and conservative ideology [28].

Culturally appropriate interventions that focus on tailored messaging and offer multiple strategies that are low cost and easily accessible were shown to have an effect in reducing infectious disease transmission among BLMSM [29, 30]. One such study found that targeted health promoting activities, such as receiving tests for sexually transmitted infections and prevention measures for HIV, were associated with higher COVID-19 vaccine uptake among MSM [31]. Conducting community outreach and promotional activities in community centers, churches, and other faith-based meeting places, as well as salons and barbershops, were also effective in increasing COVID-19–related preventive measures in targeted communities [32].

Participants for this study were part of the Harnessing Online Peer Education (HOPE) project, which seeks to determine the efficacy of using online social networks to scale peer community leader models to increase HIV prevention within BLMSM. An online survey was sent to the HOPE cohort about their intention to get a COVID-19 test, willingness to receive a COVID-19 vaccine when available, and to install a contact tracing app on their mobile device to prevent the spread of COVID-19. At the time of the study, there were a limited number of test kits available and less information about a potential vaccine that would be effective in reducing community transmission. This study aimed to evaluate the factors associated with BLMSM’s previous testing, intent to be vaccinated, and willingness to use digital contact tracing apps during the first peak of the pandemic.

## Methods

### Data Collection and Recruitment

**Fig. 1:**
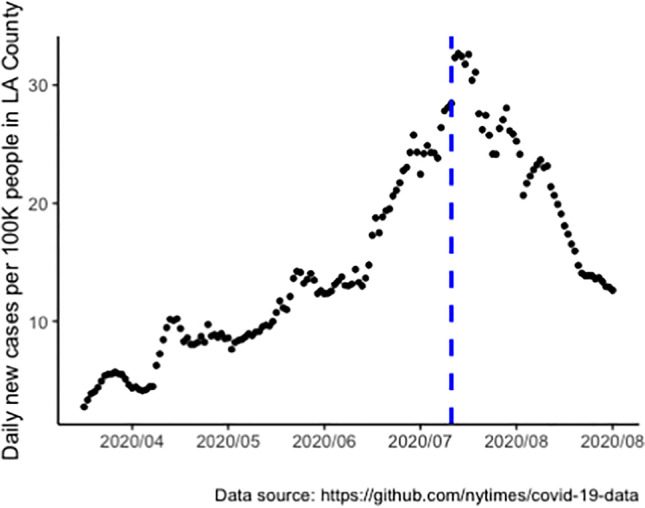
HOPE COVID-19 follow-up survey timeline and case rate of Los Angeles County

The survey was conducted at the initial height of the COVID-19 pandemic, from July 8 to July 14 in 2020 (Fig. 1). All participants were recruited directly from the ongoing HOPE cohort (NCT02944877). HOPE cohort participants were 18 years or older at enrollment, racial or ethnic minority-identified MSM, and self-reported as HIV negative, serostatus, or unknown status. Participants were randomly assigned in waves to a control or intervention arm. The intervention arm included peer-delivered HIV information in private Facebook groups. Participants were followed up at 3 months, 6 months, and 12 months post intervention. A more detailed description of the HOPE cohort study can be found in an earlier paper [33]. This COVID-19 follow-up survey recruited the first 300 participants who responded to the invitation link sent via email. The study was approved by UCLA IRB.

At the beginning of the survey, our research team explained to participants that their participation was voluntary. The decision to participate or not participate in this additional survey had no impact on their involvement in the current HOPE study. The total length of the survey took less than 1 h to complete, and an e-gift card, in the amount of $20 was offered at the end of the survey. All surveys were completed online using survey monkey. Consent to participate was asked at the start of the survey.

### Dependent Variables

The three prevention strategies to limit COVID-19 transmission, previous testing, vaccination intent, and the use of a contact tracing apps, were the dependent variables chosen for this study. To determine respondents’ receipt of a COVID-19 test, the following combination of two questions was used: (a) “Have you been tested for a COVID-19 infection?” or (b) “Has a healthcare provider ever told you that you had COVID-19?” each with the binary answer of “yes” or “no.” Since the COVID-19 vaccination was not available at the time of the survey, vaccination intent was measured by the question: “Once a vaccine for COVID-19 is manufactured, how likely are you to get the vaccine?”. This question was asked at the first peak of cases rose in Los Angeles, before a vaccine was developed, before the effectiveness of vaccines and their side effects were known. The willingness to use a contact tracing app was coded in response to the question “How likely would you be to download and use a contact tracing app or wearable that tracks your location, who you interact, and reports the data to researchers/health departments trying to help stop the spread of COVID-19?”. A five-point Likert scale of responses was used for both questions: “extremely,” “very,” “moderately,” “slightly,” and “not at all.” The answers were collapsed from a 5-item Likert scale of “Extremely” to “not at all” into a binary variable of “yes” (“Extremely” and “Very”) and “no” (“Moderately,” “Slightly,” and “Not at all”).

### Independent Variables

#### HIV Intervention Participation

Intervention participation was measured by participants either being involved with peer-delivered HIV information in private Facebook groups (intervention arm) or were a part of the control group, which did not receive this intervention. **Binge drinking** was defined by consuming four or more drinks on a typical day over the previous 3 months. A gender-neutral standard drink is about one small glass of wine (5 oz, 12% alcohol), one beer (12 oz, 5% alcohol), or one single short of liquor (1.5 oz, about 40% alcohol), according to the National Institute on Alcohol Buse and Alcoholism (NIAAA). **Depression** was measured by the Beck Depression Inventory (BDI), which includes 21 questions each on a 4-point scale (0,1,2,3) [34]. The sum of all scores ranged from 0 to 63 with higher score indicating higher level of depression. In non-clinical populations, depression is defined as score 20 and above. **Anxiety** was assessed by Generalized Anxiety Disorder 7-item (GAD-7) scale [35]. It contained 7 questions, with the summed scale ranging from 0 to 21. A threshold of 10 on the GAD-7 was used to screen for anxiety disorder.

Both the Intervention and Control subjects were clustered in private Facebook groups. We conducted bivariate analysis with chi-square test and multivariate analysis using logistic regression with these Facebook private groups as a clustered random effect. In the regression model, we included factors such as intervention, age, education, marital status, employment status, general health, and mental health indicators like depression and anxiety. We also considered behavior-related health factors, such as tobacco use and instances of binge drinking. We checked for collinearity among the covariates—binge drinking, tobacco use, depression, and anxiety—using regression analysis. We used a condition index < 30 as the cutoff for no collinearity. All these variables met the criteria. We reported odds ratio and its 95% confidence interval. All statistical analysis was conducted using SAS 9.4.

## Results

### Characteristics of the Sample

A comparison of baseline characteristics was conducted between those who responded to the follow-up COVID-19 survey and those who did not respond to the follow-up survey within the HOPE cohort. There were no significant differences between the two groups, except for higher educational level (at least an Associate’s or Bachelor’s degree) of the COVID-19 survey respondents. Educational attainment was included in the analyses of this study.

#### Sample Demographics

The mean age of participants (*N* = 300 out of 720) was 33.7 years ranging from 21 to 65 years as shown in Table 1. Two-thirds of the sample had lived in Los Angeles for more than 2 years, while a third moved in the city within the past 2 years at the time of recruitment. A majority (63%) of the participants were Latinx, had a college degree (77%), and were single (70%). About half of the participants (57%) worked full time, working more than 35 h per week, in the past month. A third of the participants (33%) identified as an essential worker in Los Angeles. During the lockdown, essential workers were exempt from stay-at-home and shelter-in-place orders, and were required to report to their workplace. Essential workers included but were not limited to those working in public health or health care, law enforcement, public safety, first responders, food and agriculture, energy and electricity, petroleum, water and waste, transportation, public works, and communications. Approximately half (*n* = 147, 49%) of the participants were able to work remotely.

**Table 1:** Characteristics of the participants

Variables	Statistics (*N* = 300)
Age	33.7 (8.9), range 21–65
Intervention	
Control	144 (48)
Intervention	155 (51.7)
Race/Ethnicity	
White/European descent	0 (0)
Black/African American	53 (17.7)
American Indian or Alaska Native	11 (3.7)
Asian or Pacific Islander	18 (6)
Latino/Caribbean	188 (62.7)
Other	30 (10)
Education	
High school or less	70 (23.3)
Associate’s or Bachelor’s degree	154 (51.3)
Graduate and above	76 (25.3)
Marital status	
Single (never married)	233 (77.7)
Married or domestic partnership	91 (30.3)
Current work	
Full time (35 + h/week)	171 (57)
Part time (< 35 h/week)	88 (29.3)
Not working	41 (13.7)
Essential worker	
Yes	99 (33)
Not working	88 (29.3)
No	113 (37.7)
Tobacco (past 3 months)	
Yes	75 (25)
No	225 (75)
Binge Drink (4 + drinks on a day)	
Yes	141 (47)
No	159 (53)
Substance use	
Not use any	136 (45.3)
Marijuana	148 (49.3)
Other	16 (5.3)

#### Health-Related Behaviors

About 25% of the participants used tobacco in the past 3 months. Nearly half (47%) of the participants indicated having four or more drinks at least 1 day in the past 3 months. Substance use (5%) was not common among participants if marijuana use was excluded. Table 2 presented the health-related outcomes in this study. About half (48%) of the participants reported that they were healthy, but approximately a third of participants had depression (23%) and anxiety (31%). There were 42% participants who tested for COVID-19 in the past 3 months, 11 of whom confirmed a positive diagnosis. About a third (27%) did not want to be vaccinated should it become readily available. Nearly half of the participants (45%) were willing to use digital contact tracing apps.

**Table 2:** Health-related outcomes and COVID-related outcomes

Variables	*N* (%)
General health	
Healthy	143 (47.7)
Unhealthy	45 (15)
Average	112 (37.3)
Depression	
Yes	69 (23)
No	231 (77)
Anxiety	
Yes	92 (30.7)
No	208 (69.3)
Test for COVID-19	
Yes	125 (41.7)
No	175 (58.3)
Results of the test	
Positive	11 (3.7)
Negative	289 (96.3)
Vaccine for COVID-19	
Yes	219 (73)
No	81 (27)
Test or vaccine	
Yes	247 (82.3)
No	53 (17.7)
Install COVID-19 app	
Yes	136 (45.3)
No	164 (54.7)

In Table 3, we use Chi-square test to estimate the association between each covariate variable and each prevention strategy respectively (1) tested for COVID-19; (2) vaccination intent; (3) either test for COVID-19 or vaccination intent; (4) willingness to use digital contact tracing apps. BLMSM participants with a graduate degree or above were more likely to use all three strategies. In particular, participants who earned a graduate degree or above were almost twice likely to use digital contact tracing apps than those with a college degree or less (*p* < 0.001). Those married or living with a partner, employed, and not depressed were more likely to use these prevention strategies. Additionally, BLMSM who had no depression were more likely to have previously tested for COVID-19 (*p* = 0.04).

**Table 3:** Relationship between factors and tested for COVID, vaccination intent, both tested for COVID and vaccination intent, and willingness to use digital contact tracing apps

	Tested for COVID	Vaccination intent	Combine both	Willingness to use digital tracing apps
Characteristics	***N* (%)**	***N* (%)**	***N* (%)**	***N* (%)**
Intervention				
Intervention	64 (41)	118 (75.6)	128 (82.1)	73 (46.8)
Control	61 (42.4)	101 (70.1)	119 (82.6)	63 (43.8)
Education				***
High school or less	24 (34.3)	48 (68.6)	54 (77.1)	18 (25.7)
Associate’s or Bachelor’s degree	66 (42.9)	109 (70.8)	126 (81.8)	73 (47.4)
Graduate and above	35 (46.1)	62 (81.6)	67 (88.2)	45 (59.2)
Marital status				
Single (never married)	96 (41.2)	165 (70.8)	187 (80.3)	103 (44.2)
Married or in partnership	29 (43.3)	54 (80.6)	60 (89.6)	33 (49.3)
Current work				
Full time (35 + h/week)	72 (42.1)	127 (74.3)	142 (83)	82 (48)
Part time (< 35 h/week)	19 (46.3)	30 (73.2)	36 (87.8)	20 (48.8)
Not working	34 (38.6)	62 (70.5)	69 (78.4)	34 (38.6)
General health				
Healthy	66 (46.2)	96 (67.1)	112 (78.3)	66 (46.2)
Average	41 (36.6)	86 (76.8)	96 (85.7)	52 (46.4)
Unhealthy	18 (40)	37 (82.2)	39 (86.7)	18 (40)
Binge drink (4 + drinks)				
Yes	53 (37.6)	105 (74.5)	113 (80.1)	66 (46.8)
No	72 (45.3)	114 (71.7)	134 (84.3)	70 (44)
Tobacco (past 3 months)				
Yes	35 (46.7)	53 (70.7)	60 (80)	34 (45.3)
No	90 (40)	166 (73.8)	187 (83.1)	102 (45.3)
Depression	*		*	
Yes	21 (30.4)	49 (71)	51 (73.9)	31 (44.9)
No	104 (45)	170 (73.6)	196 (84.8)	105 (45.5)
Anxiety				
Yes	35 (38)	71 (77.2)	76 (82.6)	42 (45.7)
No	90 (43.3)	148 (71.2)	171 (82.2)	94 (45.2)

*< 0.05

**< 0.01

***< 0.001

Table 4 presents the multivariate analysis, reporting adjusted odds ratios and 95% confidence intervals. Overall, participants who were older, had a higher level of education, married or living with a partner, employed, not depressed, or indicated being anxious were found to have higher odds of using these prevention strategies, with odds ratio greater than 1. Controlling for intervention participation, age, education, marital status, employment, health, tobacco, binge drinking, and self-reported anxiety, those who were depressed had 33% (95% CI: 0.13 to 0.82) odds of using a prevention strategy (either test for COVID-19 or vaccination intent) as the group who were not depressed. Those who had high school diploma or less had 23% (95% CI: 0.11 to 0.48) odds to use digital contact tracing apps as the group with education level of at least Associate’s or Bachelor’s degree.

**Table 4:** Odds ratio (95% confidence interval) for COVID test, vaccine, test or vaccine, and COVID app installation

	Tested for COVID	Vaccination intent	Combine both	Willingness to use digital tracing apps
Intervention				
Yes (reference)				
No	1.01 (0.61,1.66)	0.68 (0.39,1.21)	1.03 (0.53,1.99)	0.86 (0.52,1.42)
Age				
One year increase	1 (0.97,1.02)	1 (0.97,1.03)	1 (0.96,1.04)	1.02 (0.99,1.05)
Education				
High school or less	0.59 (0.29,1.23)	0.52 (0.23,1.20)	0.47 (0.18,1.24)	0.23 (0.11,0.48)***
Associate’s or Bachelor’s degree	0.86 (0.48,1.56)	0.53 (0.26,1.08)	0.56 (0.24,1.33)	0.6 (0.33,1.09)
Graduate and above (reference)				
Marital status				
Married	1.01 (0.56,1.84)	2.05 (0.99,4.25)	2.19 (0.87,5.49)	1.09 (0.59,1.99)
Single (reference)				
Employment				
Other	0.99 (0.56,1.75)	0.86 (0.46,1.62)	0.93 (0.45,1.90)	0.76 (0.43,1.34)
Part time	1.35 (0.64,2.85)	1.08 (0.47,2.49)	1.78 (0.61,5.19)	1.24 (0.60,2.64)
Full time (reference)				
General health				
Unhealthy	0.93 (0.44,1.97)	2.66 (1.07,6.64)*	2.78 (0.96,7.99)	0.79 (0.37,1.68)
Average	0.7 (0.41,1.19)	1.67 (0.92,3.04)	1.87 (0.91,3.84)	1.02 (0.60,1.74)
Healthy (reference)				
Binge drink (4 + drinks)				
Yes	0.72 (0.43,1.21)	1.28 (0.72,2.27)	0.87 (0.45,1.71)	1.2 (0.72,2.01)
No (reference)				
Use tobacco				
Yes	1.75 (0.96,3.17)	0.9 (0.47,1.73)	0.96 (0.45,2.04)	1.14 (0.63,2.07)
No (reference)				
Depression				
Yes	0.47 (0.22,0.99)*	0.55 (0.25,1.23)	0.33 (0.13,0.82)*	1.17 (0.57,2.41)
No (reference)				
Anxiety				
Yes	1.12 (0.59,2.10)	1.88 (0.90,3.94)	1.78 (0.75,4.24)	1.12 (0.60,2.10)
No (reference)				

*< 0.05

** < 0.01

***< 0.001

## Discussion

In this paper, we evaluated three prevention strategies, previous testing, vaccination intent, and willingness to use digital contact tracing apps, that were implemented to reduce the spread of COVID-19 at the early stage of pandemic in Los Angeles. This study aimed to estimate the demographic factors, health-related behaviors, and other characteristics that could be associated with these prevention strategies, particularly during a time when there was limited knowledge about the COVID-19 virus, there were not enough COVID-19 test kits, an effective vaccine had not been developed, and there were no fully developed digital contact tracing apps to protect oneself from acquiring the virus. We modeled each potential prevention strategy to better understand how a particular priority community, BLMSM, may use these strategies to reduce COVID-19 transmission. Our study found that BLMSM who were depressed (with a BDI score of more than 20 compared to the general population) had lower odds of having tested and had an intent to vaccinate for COVID-19. Those who reported being unhealthy were more likely get a hypothetical vaccine. Additionally, BLMSM who received or had less than a high school degree had lower odds of being willing to use digital contact tracing apps compared to those who had completed graduate school or above.

### Factors Associated with COVID-19 Testing among BLMSM

Our study found that 42% of participants had a COVID-19 test in the last 3 months at the first peak of the pandemic—a result that is consistent with previous studies conducted among sexual and gender minorities (SGM). Approximately 38.3% of LGBT [13] US residents and 44.5% of MSM in Australia [36] indicated having taken a COVID-19 test in the summer of 2020. Higher levels of depression were also shown to negatively influence COVID-19 testing among BLMSM in this study. Indeed, several studies have demonstrated the psychological toll the gay and bisexual MSM community bore during pandemic, and in particular those who experienced financial, housing, and food insecurity during this time [11, 37, 38]. Although less is known about how higher depression levels may impact testing for COVID-19 among BLMSM, previous studies demonstrated that depression and other mental health disorders negatively influence gay and bisexual MSM healthcare seeking behavior, such as HIV testing uptake [39, 40] . Additionally, the pandemic placed many within the gay and bisexual MSM community at higher risk for economic instability, while reducing their ability to access reliable health and social services—which may have further limited their chances to receive a COVID-19 test [11, 38].

### Factors Associated with COVID-19 Vaccination Intent among BLMSM

.With the arrival of an effective vaccine, the focus of COVID-19–related research has shifted from reducing the virus’ health impact to vaccine hesitation among different groups, long COVID-19 symptoms, and economics recovery [41–44]. Previous studies demonstrate that COVID-19 related vaccine hesitancy is largely due to misinformation spread regarding vaccine safety and its effectiveness, along with historical institutional mistrust, particularly within marginalized communities [45, 46]. However, our study results show that only 27% of participants had low intention of getting vaccinated for COVID-19 once it became available. This is consistent with nationwide surveys conducted in the USA which demonstrate SGM individuals’ high COVID-19 vaccination coverage. By October 2021, approximately 85.4% of SGM individuals reported having completed the recommended COVID-19 vaccination regimen [47]. Only those who identified as non-Hispanic Black SGM individuals had a lower vaccination rate at 69.7% [47]. A majority of gay and bisexual MSM indicated higher than average confidence in the COVID-19 vaccine, at 82.4% and 76.3%, respectively [47].

In addition, our study found that a higher depression level was associated with low vaccination intent among BLMSM [24, 48–50]. Current publications show mixed results with some studies indicating that a higher depression level was not significantly associated with vaccine acceptance, [51–53] while other studies suggest that mental health disorders, including depression, may increase vaccine hesitancy [50, 54]. Finally, our study found that self-reported poor health was associated with higher vaccination intent among BLMSM. This result differentiated from a cross-sectional global study conducted in January 2021 wherein individuals with comorbid conditions, including cancer, autoimmune disease, and chronic lung disease, continued to be hesitant about the safety and efficacy of the COVID-19 vaccine [55]. However, others studies conducted particularly among MSM indicate that perceived susceptibility to COVID-19 were associated with a higher intent to be vaccinated [55, 56]. Other factors influencing MSM’s vaccination hesitancy worldwide include younger age, lower income, lower education and health literacy level, and rurality [56, 57]. These factors were not consistent over the course of COVID-19 changes, which indicated that the prevention struggles of pandemic and promoting vaccine straggles should be adjusted accordingly [57].

### Factors Associated with Willingness to Use Digital Contact Tracing Apps

We found that participants who had a high school education or less were had lower odds of being willing to use digital contact tracing apps, even hypothetically, which is consistent with current literature [58, 59]. This result may need further investigation as gay and bisexual MSM, and BLMSM in particular, regardless of educational level, have long used emerging technology to improve their health outcomes [60, 61]. Our study however, did not evaluate participants’ ethical or privacy concerns about installing the digital contact tracing app at the time of the survey which may have influenced their response. Once these digital contact tracing apps and products were released and used by the public, ethical guidelines and confidential concerns became apparent, particularly with devices and apps that track participants’ locations and health information [62, 63].

## Limitations

The results from this study should be generalized with caution, given the timing of our data collection regarding willingness to use a vaccine and install an app to trace COVID-19. Both the vaccine and the mobile app were not available at the time of the study. Study participants had just recovered from the strict shelter-in-place order. We dichotomized the responses from a 5-level Likert scale aiming to find the association of willingness to get vaccinated and install the app with extremely likely scenarios. This approach may result in the loss of information from the full Likert scale. Additionally, causal inference and the direction of the associations described in this study cannot be established since the data were derived cross-sectionally. This data is also based on self-report which can be vulnerable to recall bias and misclassification of the study’s outcomes.

Another limitation of the study is the smaller sample size nested within the HOPE cohort. The participants were selected based on their early response to the survey request. The sample size is pre-determined at a cutoff of 300. There was no randomization process involved though the final distribution was even in the HOPE cohort across five waves and across intervention and control arms. The cross-sectional analysis may not reflect the change among MSM individuals during different waves of the pandemic, even within the HOPE cohort. It is a snapshot of potential associations between prevention strategies and characteristics of the survey participants. In this study, we did not include the social determinants of health that may impact the decision on prevention strategies. Potential uncontrolled confounding factors should be considered for the generalization of the study.

Extra caution is needed when interpreting the impact of an intervention through social media. Under shelter-in-place orders, people generally spend more time at home and online. Social media intervention may not work as designed. In the ongoing trial, we have altered some recruiting and training methods. For example, during the latest wave, the peer-leader training happened outdoors instead of indoors. In 2020, people dropped out of study due to uncomfortable election conversations.

## Conclusion

We evaluated three strategies to prevent the COVID-19 transmission and found that depression among BLMSM, a high priority population, may be associated with lower testing and intent to vaccinate for COVID-19. Additionally, having a high school education or less indicated lower odds of wanting to use digital contact tracing apps in this population. To help support public health programs seeking to improve uptake of prevention strategies of novel infectious diseases, particularly among BLMSM, various measures are recommended. First, Black and Latinx as well as sexual and gender minority communities face intersecting barriers to care. Improving access to health services, and mental health services in particular, should be an essential part of any public health strategy intended to improve testing and vaccination uptake within these marginalized communities. Additionally, promoting programs on digital literacy is recommended for BLMSM who have a high school education or lower to improve their willingness to use digital contact tracing apps. The intention to get vaccinated and use a contact tracing app was hypothetical at the beginning of the pandemic. Making contact tracing app user-friendly and paying extra attention to app users with varied needs may help improve the vaccination rate in this population at the onset of a pandemic. Finally, it is important to note that culturally appropriate interventions, informed by community members themselves, have long been found to positively influence preventive behaviors among BLMSM. Public health testing and vaccination programs that are conducted in the community such as churches, community centers, salons, and barbershops, should continue as they may reach subsets of the BLMSM community.

## Data Availability

Data will be made available upon request to ensure the privacy of the study participants.

## Code Availability

Code will be available upon request to corresponding author.
